# Chloroform Administration

**Published:** 1902-04-26

**Authors:** 


### Chloroform Administration.

An elaborate investigation lately undertaken by Dr. Embley,[1] honorary anæsthetist to the Melbourne Hospital, into the causation of death during the administration of chloroform throws much light upon the origin of these sad accidents, for by a series of extremely ingenious and carefully conducted experiments he has succeeded in giving such a clear and coherent picture of what happens during the production of anæsthesia as to indicate with no uncertain hand where danger lies. Naturally a good deal hinges upon the question how far the findings of the Hyderabad Commission as to the relation between cardiac and respiratory failure are to be accepted, or indeed are germane to the problem at all, and in regard to the latter point it seems clear that, however completely and accurately the Commission may have described the succession of events in gradually deepening narcosis, their records are imperfect in regard to the very early stages of anæsthesia. Dr. Embley says "their dogs were generally anæsthetised by chloroform in a box" before being brought under observation. "The graphic records of the Commission therefore only represent the comparatively safe period of chloroform administration in dogs. The Commission were unable to inform us concerning the interesting phenomena that occurred in the box."

To clinicians, however, it is just what happened "in the box" that is of the chiefest interest, for it is at that period, just at the "going under" stage, that the most tragic accidents occur. Man, like any animal, may of course be "chloroformed to death" if the administration be carried too far or continued too long. But such an accident is a gradual affair, and when the symptoms occur one knows what to do. The horrible cases, those in which the heart suddenly stands still and refuses to start again, do what we may, often occur quite at an early stage, and some of the most striking of Dr. Embley's experiments are those by which he shows what takes place, if one may so say, "in the box," i.e., during the dangerous period of on-coming anæsthesia. He shows among other things (1) that the heart muscle is very sensitive to the poisonous effects of chloroform, emphatically denying the assumption of the Hyderabad Commission that chloroform by inhalation never impairs the heart; and (2) that the effect of chloroform upon the nerve centre, particularly in the early part of the administration, is to raise the excitability of the vagus mechanism by which the heart's action is inhibited. It is on this vagus inhibition that he throws the chief blame for sudden death in the early stages of administration, the inhibition being all the more intense and fatal in its effect from its being exercised at a moment when the heart muscle has already been weakened by the action of the chloroform upon it. Thus the idea which one gains from these various experiments as to what happens during chloroform administration is that from the moment the drug reaches the heart the heart muscle begins to be paralysed; that meanwhile the blood pressure falls, partly in consequence of this paralysis of the heart and partly also from paralysis of the arterioles; and that as a further effect of this fall in blood pressure failure of respiration may occur. During the early stage of administration, however, another event has been taking place which is of still greater importance, namely, an excitation of the vagus mechanism, and it is to this that sudden stoppage of the heart at this stage is due. By degrees this excitability passes off, but so long as it continues there is danger of the heart being brought to a stand, and what must be remembered is that it depends largely upon the strength of the chloroform vapour which is used. A weak vapour produces only a mild excitation followed by a depression of vagus irritability, while on the other hand a strong vapour at once irritates the vagus mechanism and sets up a strong inhibitory effect. Thus we come round to the old story that for safety in chloroform administration only a very weak vapour must be employed. Dr. Embley says, "Use only weak vapour of chloroform (less than 1 per cent.) in the early stages, until the initial increased excitability of the vagus mechanism has given place to diminished excitability; in other words, take time in putting the patient under." What the influence of these investigations will be upon the current teaching in regard to chloroform administration one hesitates to say. Anæsthetists have shown themselves curiously obstinate and retentive of traditional methods; but it seems very clear that, if Dr. Embley's experiments upon dogs are to be taken as indicating the course of events in human beings, it will become our duty to throw aside all preconceived ideas as to the safety of towels, masks, drop-bottles, and all such rough-and-ready methods of producing chloroform vapour and appeal to the mechanicians to supply us with an apparatus for use during the early stages of anæsthesia which shall by positive measurement and with unerring exactitude supply a mixture of air and chloroform vapour containing not more than 1 per cent. by volume of the latter. We doubt if such an apparatus exists at the present time.

[1] Brit. Med. Journ., April 5, 12, and 19.

